# Dependence of NMDA/GSK-3β Mediated Metaplasticity on TRPM2 Channels at Hippocampal CA3-CA1 Synapses

**DOI:** 10.1186/1756-6606-4-44

**Published:** 2011-12-21

**Authors:** Yu-Feng Xie, Jillian C Belrose, Gang Lei, Michael Tymianski, Yasuo Mori, John F MacDonald, Michael F Jackson

**Affiliations:** 1 Molecular Brain Research Group, Robarts Research Institute, University of Western Ontario, London, ON, N6K 5K8, Canada; 2 Department of Physiology and Pharmacology, University of Western Ontario, London, ON, N6K 5K8, Canada; 3 Department of Anatomy and Cell Biology, University of Western Ontario, London, ON, N6K 5K8, Canada; 4 Department of Physiology, University of Toronto, Toronto, ON, M5S 1A8, Canada; 5 Toronto Western Hospital Research Institute, Toronto, ON, M5T 2S8, Canada; 6 Department of Synthetic Chemistry and Biological Chemistry, Graduate School of Engineering, Kyoto University, Katsura Campus, Nishikyo-ku, Kyoto 615-8510, Japan

**Keywords:** TRPM2, GSK-3β, PSD-95, Long term depression, Metaplasticity, NMDA Receptors, AMPA Receptors

## Abstract

*Transient receptor potential melastatin 2 (TRPM2) is a calcium permeable non-selective cation channel that functions as a sensor of cellular redox status. Highly expressed within the CNS, we have previously demonstrated the functional expression of these channels in CA1 pyramidal neurons of the hippocampus. Although implicated in oxidative stress-induced neuronal cell death, and potentially in neurodegenerative disease, the physiological role of TRPM2 in the central nervous system is unknown. Interestingly, we have shown that the activation of these channels may be sensitized by co-incident NMDA receptor activation, suggesting a potential contribution of TRPM2 to synaptic transmission. Using hippocampal cultures and slices from TRPM2 null mice we demonstrate that the loss of these channels selectively impairs NMDAR-dependent long-term depression (LTD) while sparing long-term potentiation. Impaired LTD resulted from an inhibition of GSK-3β, through increased phosphorylation, and a reduction in the expression of PSD95 and AMPARs. Notably, LTD could be rescued in TRPM2 null mice by recruitment of GSK-3β signaling following dopamine D2 receptor stimulation. We propose that TRPM2 channels play a key role in hippocampal synaptic plasticity*.

## Background

The transient receptor potential melastatin 2 channel (TRPM2) is a novel non-selective cation channel that was initially cloned from the brain, and was subsequently identified as an effector of calcium fluxes following oxidative stress[1]. Functionally, TRPM2 has been linked to cell death, cytokine production, and insulin secretion[1]. Interestingly, TRPM2 expression is greatest in the central nervous system (CNS) where it may contribute to neurodegenerative disease[1,2]. We recently demonstrated functional expression of TRPM2 in CA1 pyramidal neurons[3], in culture and *in situ*. TRPM2 is unique among known ion channels in that it contains a cryptic C-terminal enzyme domain homologous to the NUDT9 ADP-ribose (ADPR) hydrolase. This channel motif serves primarily as the ligand binding domain for ADPR, which is required for Ca^2+^-dependent gating of the channel[4]. In hippocampal neurons, TRPM2 currents can be activated by voltage-ramps that generate inward Ca^2+ ^currents or by strong stimulation of NMDA receptors (NMDARs)[3]. Indeed, high concentrations of ADPR are unable to evoke these currents until activated by an influx of Ca^2+^. The coupling of channel activity to Ca^2+ ^signaling downstream of voltage-gated Ca^2+ ^channels and NMDARs suggests that TRPM2 could play a role in neuronal signaling or synaptic transmission. Given the lack of selective antagonists, we have examined the hypothesis that these channels contribute to synaptic plasticity by employing TRPM2 deficient mice[5].

## Results

**Figure 1:**
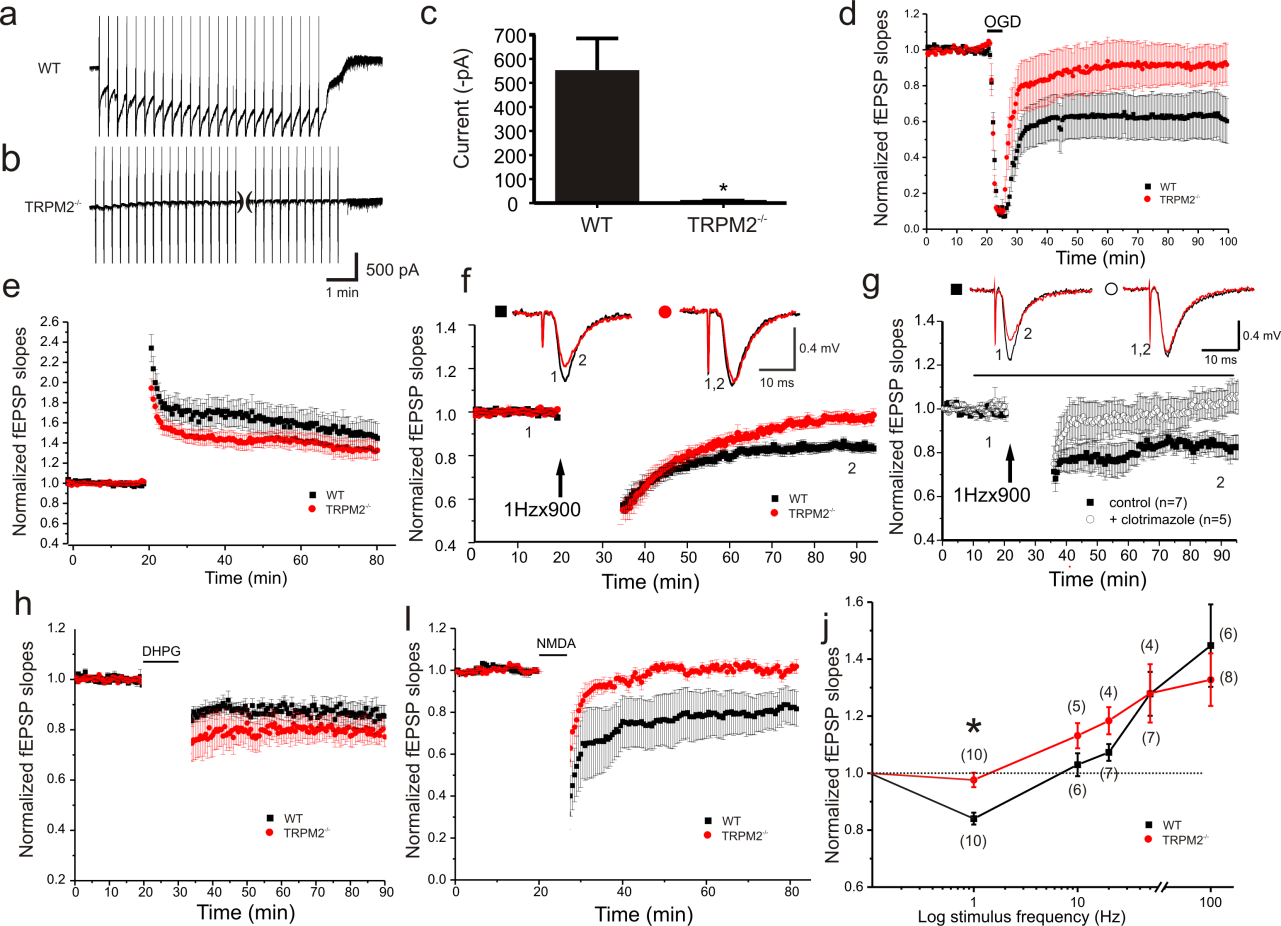
**LTD is impaired in hippocampal slices derived from TRPM2^-/- ^mice**. (a,b) ADPR-primed TRPM2 currents are facilitated by voltage ramps ( ± 100 mV, 1/10 sec) in WT (a), but not in cultured neurons from TRPM2^-/- ^mice (b). (c) Summary bar graph of ADPR-primed peak current amplitude in WT and TRPM2^-/- ^neurons. (unpaired t-test *p = *0.001, 553.4 ± 132 pA in WT, n = 5; 7.9 ± 7.2 pA in KO, n = 6) (d) Transient oxygen-glucose deprivation (5 min OGD) causes long-lasting depression of fEPSP slopes in slices from WT (*n *= 12) but not TRPM2^-/- ^(*n *= 6). (e) LTP is unaffected by knockout of TRPM2 (WT, *n *= 6; TRPM2^-/-^, *n *= 8). (f) LTD of fEPSPs evoked by repetitive stimulation (900 stimuli at 1 Hz) in WT slices (*n *= 10) is absent in slices from TRPM2^-/- ^mice (*n *= 10). (g) LTD of fEPSPs was inhibited by application of clotrimazole, a TRPM2 inhibitor. Timing of clotrimazole application, in treated slices, is indicated by the black bar. (h) Metabotropic-glutamate receptor dependent LTD is unimpaired by deletion of TRPM2 (WT, n = 6; TRPM2^-/-^, n = 7). (i) Chem-LTD, evoked by 5 min application of NMDA (10 μM) is abolished in slices from TRPM2^-/- ^(*n *= 6) but not WT (*n *= 6) slices. (j) Summary graph for a series of recordings from WT and TRPM2^-/- ^slices in which plasticity was induced by repetitive stimulation delivered at 1, 10, 20, 50 Hz (900 pulses at each frequency) or 100 Hz (four 1 sec trains delivered 20 sec apart). For each induction frequency, normalized fEPSP slopes, measured at the end of recording from each of the two populations of slices, are plotted. Numbers in parentheses represents the number of recordings at each point. *denotes p = 0.016.

**Figure 2:**
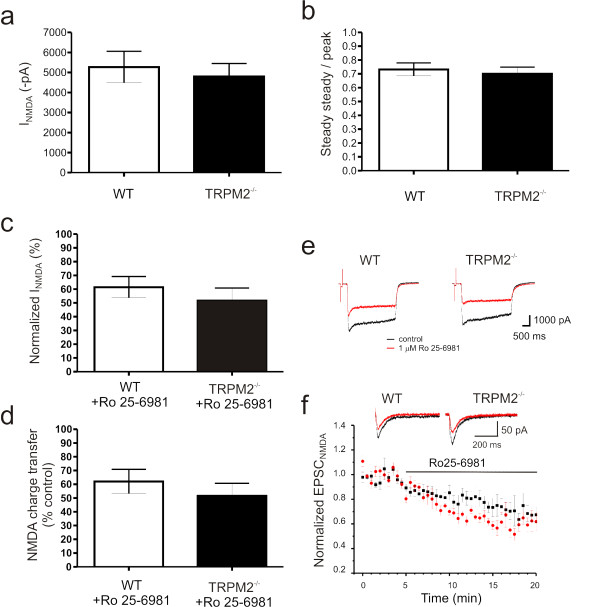
**NMDAR function is unaffected by disruption of TRPM2 channel expression**. Neither peak (a) nor steady-state/peak (b) currents, generated in response to rapid applications of NMDA to cultured hippocampal neurons, are affected by loss of TRPM2 expression. Similarly, the sensitivity of NMDA-evoked responses (peak amplitude (c), total charge transfer (d) and representative traces (e) recorded from cultured hippocampal neurons, to inhibition by the GluN2B specific antagonist, Ro 25-6981, is unaltered in neurons from TRPM2^-/-^. (f) Inhibition of pharmacologically isolated NMDAR-mediated synaptic currents, recorded from CA1 pyramidal neurons in response to Schaffer-collateral stimulation, by Ro 25-6981 is unaltered in slices from TRPM2^-/-^. For all results presented in panels a-d, *n *= 5; for panel f, WT: *n *= 6; TRPM2^-/-^: *n *= 5)

Hippocampal neurons were cultured from wildtype (WT) and knockout (TRPM2^-/-^) mice using standard procedures outlined in the methods. This allowed us to compare the activation of TRPM2 currents in cultured pyramidal neurons from both genotypes. Whole-cell voltage clamp recordings were performed with 1 mM ADPR in the patch solution. Under these recording conditions TRPM2 currents were absent until multiple voltage-ramps are used to evoke these slowly developing currents. Large TRPM2 inward currents were generated at a holding potential of -60 mV in neurons from WT mice (Figure 1a,c; 553.4 ± 132 pA, n = 5) but were entirely absent in those from TRPM2^-/- ^mice (Figure 1b,c; 7.9 ± 7.2 pA, n = 6; unpaired t-test, *p *= 0.001). Importantly, no genotypic difference in peak Ca^2+ ^currents were detected (3648 ± 715 pA in WT, 2836 ± 390 pA in KO; unpaired t-test, *p = *0.3234; data not shown). Furthermore, there were no changes in peak responses (or steady-state to peak ratios) to applications of NMDA (Figure 2a,b) or in the GluN2Bmediated component of this response determined by application of 1 μM Ro 25-6981, a highly selective antagonist of GluN2B containing receptors (Figure 2c-e). These results confirm the functional identification of TRPM2 currents in hippocampal pyramidal neurons, the absence of TRPM2 currents in neurons cultured from TRPM2 deficient mice, and that the absence of TRPM2 does not alter either voltage-dependent or NMDAR-dependent Ca^2+ ^currents.

Next, acute transverse hippocampal slices were prepared from TRPM2^-/- ^and WT mice at 3-4 weeks of age using standard approaches described in the methods. Field excitatory postsynaptic potentials (fEPSPs) were recorded from CA3-CA1. We noted no difference in the paired-pulse ratios between WT and TRPM2^-/- ^mice (additional file 1). We then examined the effects of a short period of oxygen-glucose deprivation (OGD) on fEPSPs in slices from both genotypes. In WT slices, OGD resulted in a strong depression of the fEPSPs. This depression was greatly diminished in TRPM2^-/- ^slices, consistent with a phenotype of a reduced responsiveness to the oxidative stress mediated by TRPM2 channels (Figure 1d).

Given that TRPM2 currents can be activated by NMDAR stimulation we examined whether TRPM2 might contribute to NMDAR-dependent synaptic plasticity. We first examined the induction of long-term potentiation (LTP) using a standard 100 Hz stimulation. No difference in LTP was observed in slices from TRPM2^-/- ^mice (Figure 1e) suggesting that TRPM2 plays little or no role in this form of synaptic plasticity. However, NMDARs are also required for one form of long-term depression (LTD) at these synapses so we extended our studies to include low frequency stimulation.

Applying 900 pulses at 1 Hz induced LTD in WT mice (0.84 ± 0.021, *n *= 10) but failed to do so in TRPM2^-/- ^mice (Figure 1f; 0.98 ± 0.025, *n *= 10; p = 0.017). Acute applications of the TRPM2 blocker clotrimazole also blocked LTD induced using this protocol (Figure 1g). Clotrimazole did not inhibit the induction of LTP (data not shown). LTD at these synapses can also depend upon activation of group 1 mGluRs rather than NMDARs[6], we therefore examined responses to a bath exposure to NMDA (10 μM, 5 min) or DHPG (10 μM, 20 min) in slices of both genotypes. The LTD induced by DHPG was similar in WT and TRPM2^-/- ^mice (Figure 1h; 0.85 ± 0.043, *n *= 6 in WT vs 0.80 ± 0.031, *n *= 7 in TRPM2^-/- ^mice, respectively; p > 0.05). Conversely, NMDA application induced LTD in slices from WT (*n *= 6) but failed to do so TRPM2^-/- ^mice (Figure 1i; *n *= 6). When plasticity induced by a range of repetitive stimulation frequencies was examined it was clear that TRPM2^-/- ^slices demonstrated a substantial impairment in NMDA-dependent LTD (Figure 1j).

**Figure 3:**
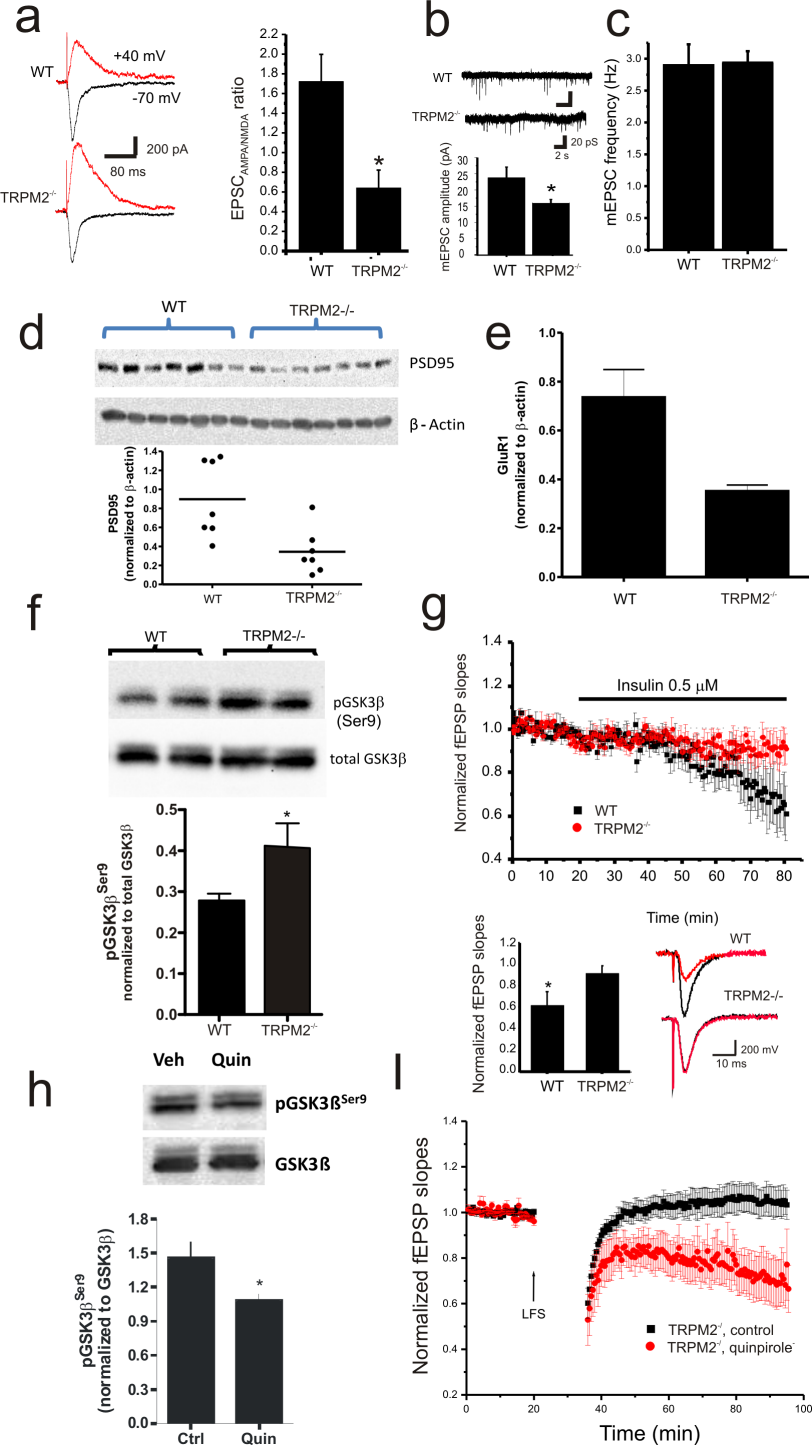
**Inhibition of GSK-3β phosphorylation and reduced PSD95 and AMPAR expression contributes to impaired LTD in TRPM2-/- mice**. (a) The ratio of AMPAR- to NMDAR-mediated EPSCs is reduced in TRPM2^-/- ^neurons. (b,c) The amplitude, but not the frequency, of mEPSCs is reduced in slices from TRPM2^-/- ^mice. Representative traces of mEPSCs recordings from slices derived from WT and TRPM2^-/- ^mice are shown. The expression of (d) PSD-95 (normalized to β-actin loading control) and (e) of the AMPAR subunit, GluR1, is depressed in slices from TRPM2^-/- ^mice. (f) Knockout of TRPM2 causes increased phosphorylation of GSK-3β, at its inhibitory site (Ser9, *n *= 3). (g) Insulin application to hippocampal slices depresses AMPAR-mediated fEPSPs in slices from TRPM2^-/-^, but not WT, slices. (h) Simulation of dopamine D2 receptors by quinpirole (10 μM) reduces GSK-3β phosphorylation at Ser9 (*n *= 3). (i) D2 receptor stimulation by quinpirole rescues LTD in slices from TRPM2^-/- ^mice.

The expression of NMDAR-dependent LTD at CA3-CA1 synapses requires the endocytosis of AMPA Receptors (AMPARs)[6,7]. To determine if there was a change in the functional expression of AMPARs in TRPM2^-/- ^mice we used whole cell voltage clamp recordings to measure the ratio of AMPAR to NMDAR mediated EPSCs. By holding the membrane potential at -70 and +40 mV the relative contribution of each receptor type was determined. This ratio was significantly reduced in TRPM2^-/- ^(*n *= 10) neurons compared to WT (*n *= 10, *p *< 0.01; Figure 3a). These results suggest that a reduction of synaptic AMPARs or alternatively an enhancement of NMDARs may underlie the deficiency of LTD in TRPM2^-/-^mice. A change in AMPARs was supported by our observation of a reduction of mEPSC amplitude but not frequency in TRPM2^-/- ^mice (Figure 3b,c). We also examined the relative contribution of NMDARs composed of GluN2A or GluN2B subunits to synaptic currents by measuring EPSC_NMDAR _before and after application of the selective GluN2B antagonist, Ro 25-6981. The relative block was the same regardless of genotype (Figure 2f).

**Figure 4:**
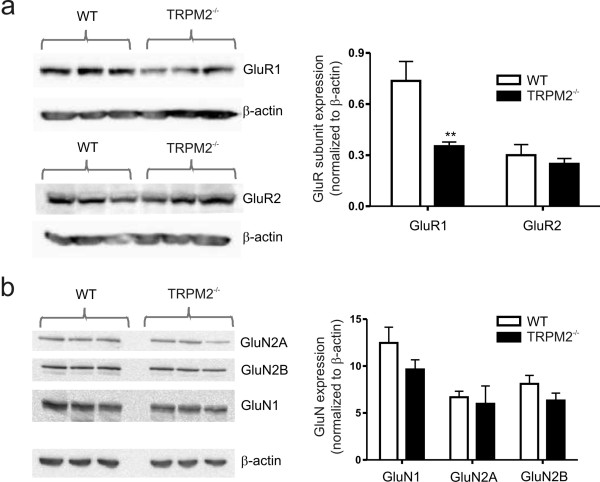
**Comparison of glutamate receptor subunit expression in hippocampal slices derived from WT and TRPM2^-/- ^mice**. (a) Western blots and summary bar graph representing the expression levels of the AMPA receptor subunit subtypes, GluR1 and GluR2. Note that the quantified data for GluR1 is the same as that presented in Figure 2. (b) Western blots and summary bar graph representing the expression levels of the NMDA receptor subunit subtypes, GluN1 (*n *= 3), GluN2A (*n *= 5) and GluN2B (*n *= 3).

During LTD there is some loss of postsynaptic density 95 protein (PSD-95) from the synapses followed by endocytosis of AMPAR[8]. Therefore, we used western blotting to examine if there was a change in PSD-95 expression in TRPM2^-/- ^mice. Consistent with the change in AMPARs, we observed a significant reduction in PSD-95 expression (Figure 3d). Previous reports link the level of PSD-95 expression with AMPAR number without an overall change in synaptic number[8,9]. We examined the total expression of GluR1 in TRPM2^-/- ^slices versus those from WT mice, and as anticipated observed a reduction in the expression of GluR1 (Figure 3e). In contrast, the expression levels of GluR2 and NMDAR subunits were the same regardless of genotype (Figure 4a,b).

To further explore the mechanism of the loss of NMDAR-dependent LTD in TRPM2^-/- ^mice we examined the activity of glycogen synthase kinase-3β (GSK-3β), which is required for this form of LTD[10,11]. We compared the expression levels as well as the phosphorylation of this kinase at Ser9, which is indicative of inactivation of GSK-3β[12]. Although the expression of GSK-3β was unaltered, there was a significant increase in Ser9 phosphorylation (GSK-3β inactivation) in TRPM2^-/- ^slices (Figure 3f) suggesting that impaired LTD in TRPM2^-/- ^mice results from inhibition of GSK-3β[10]. Insulin also acts via stimulation of Akt/PKB to inactivate GSK-3β via Ser9 phosphorylation, resulting in a long lasting depression of AMPAR-mediated synaptic transmission, which in turn occludes low frequency induced LTD[13]. We therefore examined the effect of insulin on wildtype and TRPM2^-/- ^slices. The long-lasting depression induced by applications of insulin was lacking in TRPM2^-/- ^slices (Figure 3g) suggesting that insulin-induced LTD was occluded in this genotype due to the pre-existing inactivation of GSK-3β. To determine if the loss of LTD in TRPM2^-/- ^was attributable to the consistent inhibition of GSK-3β we applied the D2 agonist, quinpirole in order to stimulate Akt GSK-3β signaling[14] and observed an enhanced activation of GSK-3β (Figure 3h) and rescue of LTD in TRPM2^-/-^mice (Figure 3i).

## Discussion

Our results show that loss of TRPM2 expression modifies NMDAR-dependent synaptic plasticity, specifically LTD, but they do not implicate TRPM2 channels directly in excitatory synaptic transmission. Instead, the presence of TRPM2 channels is associated with maintenance of the appropriate level of GSK-3β activation as well as full expression PSD-95 and AMPARs. In the absence of the TRPM2 expression there is a reduction in the relative contribution of AMPA receptors to synaptic transmission at CA1 synapses along with a reduction of LTD and a shift to lower thresholds for the induction of LTP. This metaplastic shift towards LTP in TRPM2^-/- ^slices results from enhanced phosphorylation and inactivation of GSK-3β. The resulting inactivation of GSK-3β prevented further depression of synaptic transmission by applications of insulin. Importantly, applications of quinpirole reduced GSK-3β phosphorylation in TRPM2^-/- ^slices and effectively rescued LTD induction by repetitive low-frequency stimulation. Moreover, acute block of TRPM2 function by clotrimazole prevented LTD induction thereby mimicking TRPM2 loss of function by genetic deletion. Collectively, these findings argue that the reductions in GSK-3β activity, PSD-95 and GluR1 expression, as well as AMPAR-mediated synaptic transmission and plasticity observed in hippocampal slices derived from TRPM2^-/- ^mice cannot simply be attributed to developmental compensations resulting from genetic deletion of TRPM2. Rather, in light of our findings, we propose that ongoing TRPM2 channel activity contributes to the regulation AMPAR-mediated fast excitatory synaptic transmission through maintenance of constitutive GSK-3β activity.

The precise mechanism through which TRPM2 activity regulates GSK-3β remains to be determined; however the contribution of TRPM2 to Ca^2+ ^signaling is likely to play an important role is this regard. Previous findings have proposed the Ca^2+^-dependent phosphatase, calcineurin (or PP2B), as a candidate for mediating dephosphorylation of GSK-3β at Ser9[15]. Alternatively, the Ca^2+^-dependent tyrosine kinase Pyk2 has been shown to associate and regulate GSK-3β activity through tyrosine phosphorylation[16]. Whether the activity of either calcineurin, Pyk2, or other kinases or phosphatases are altered by TRPM2-initiated Ca^2+ ^signaling remains to be established. Similarly, the behavioural consequence of altered synaptic transmission and plasticity associated with the loss of TRPM2 function has yet to be investigated; however, a recent paper by Kim and colleagues (2011) may offer some insight. These authors demonstrated that behavioural flexibility, evaluated by reversal learning in the Morris water maze and by the delayed non-match to place T-maze task, was reduced following genetic disruption of PI3Kγ, which also resulted in a selective loss of NMDAR dependent LTD [17]. Additional research is required to assess whether these behaviours are similarly altered in TRPM2^-/- ^mice.

## Conclusions

The present study establishes that loss of TRPM2 function through genetic means provokes long lasting changes in excitatory synaptic transmission and plasticity at the CA3-CA1 hippocampal synapse. Specifically, we show that the ability to induce NMDAR-dependent LTD is impaired in TRPM2^-/- ^mice. Such impairment was associated with increased phosphorylation and inactivation of GSK-3β. Consistent with previously reported changes associated with GSK-3β inhibition, we report reduced PSD95 and GluR1 expression and identify a corresponding reduction in AMPAR-mediated synaptic transmission. Interestingly, both GSK-3β and TRPM2 channels have been implicated in a number of overlapping pathophysiological processes ranging from pancreatic insulin secretion to Alzheimer's disease[1,18]. Accordingly, our results show the importance of continuing studies to determine the pathophysiological roles of GSK-3β and TRPM2 in neurodegenerative diseases[19].

### Methods Experimental animals

TRPM2^-/- ^mice, generated as described previously[5], were provided by Dr Y Mori. Wild-type and KO mice used for experimentation were derived from heterozygous matings and mouse genotyping was performed as previously described[5]. Mice were kept in a pathogen-free environment, and analyses were performed using mice that were matched for age. All animal experiments were performed in accordance with protocols approved by the University of Western Ontario Animal Use Subcommittee of the University Council on Animal Care.

### Whole-cell recording from primary culture

Mouse hippocampal primary neuronal cultures were prepared from timed-pregnant TRPM2^-/- ^or WT mice according to previously described procedures[20]. Currents were recorded from 21-28 days in vitro. Whole-cell voltage-clamp recordings were performed as described previously[3], with minor adjustments. Briefly, standard intracellular solution (ICS) contained (in mM): 150 cesium gluconate, 10 Hepes, and 2 MgCl2. To record NMDA currents, 11 mM EGTA, 1 mM CaCl2, and 2 mM K2-ATP was added to the ICS. TRPM2 currents were recorded with 1 mM ADPR added to the ICS. Standard extracellular solution (ECS) contained (in mM): 140 NaCl, 5.4 KCl, 25 Hepes, 33 glucose, 2 CaCl2, and 0.2 μM TTX. To generate NMDA currents, 50 μM NMDA and 0.5 μM glycine was added to the standard ECS and applied for 3 seconds every minute using a multibarrelled rapid perfusion system (SF77B; Warner Instruments). For experiments examining the contribution of GluN2B receptor, 1 μM Ro 25-6981 (Tocris) was applied after having obtained 3 stable control sweeps in its absence. ADPR-primed TRPM2 currents were generated with voltage ramps ( ± 100 mV, 1/10 sec) applied in the presence of standard ECS supplemented with 1 mM MgCl2 and 1 mM BaCl2. After the TRPM2 current stabilized, or after 10 minutes of voltage ramps, calcium-free solution was applied (total divalent concentration maintained by replacing CaCl2 with equimolar BaCl2). Voltage-clamp recordings (Vhold = -60 mV) were performed at room temperature (20-22°C) using a Multiclamp 700A amplifier (Molecular Devices). Data were filtered at 2 kHz, digitized, and acquired using pCLAMP and Axoscope software (Molecular Devices).

### Hippocampal slices preparation

Hippocampal slices were prepared from age-matched TRPM2^-/- ^and wild type mice (post-natal day 20-30). Briefly, mice were decapitated after isoflurane anesthesia, and brains were quickly removed and placed in ice-cold oxygenated (95% O_2_, 5% CO_2_) artificial CSF (ACSF) containing: 124 mM NaCl, 3 mM KCl, 1.25 mM NaH2PO4, 1.3 mM MgCl2, 2.6 mM CaCl2, 26 mM NaHCO3 and 10 mM glucose (osmolality between 300 and 310 mOsm). Whole-brain coronal slices (300 μm) containing transverse sections of the hippocampus were prepared using a vibrating microtome (VT100E; Leica). After a recovery period of 1 h in oxygenated ACSF, hippocampal slices were transferred to a recording chamber continuously perfused with oxygenated ACSF (3 ml/min), warmed to 31°C through an in-line solution heater.

### Field excitatory postsynaptic potentials (fEPSP) recording from Schaffer collateral-CA1 synapses

fEPSPs were evoked every 30 s (0.033 Hz) by electrical stimulation (100 μs duration) delivered to the Schaffer-collateral pathway using a concentric bipolar stimulating electrode (25 μm exposed tip) and recorded using glass microelectrodes (3-5 MΩ, filled with ACSF) positioned in the stratum radiatum of the CA1 area. The stimuli intensity was adjusted to evoke fEPSPs corresponding to 30~50% of the maximal response evoked in the absence of a contaminating spike discharge. The paired-pulse ratio was determined in each slice by delivering pairs of stimuli at varying interstimulus intervals (10-1000 ms). For LTD/LTP experiments, baseline fEPSPs were monitored for at least 20 min to ensure stability, following which synaptic plasticity was induced by repetitive stimulation delivered at 1, 10, 20, 50 Hz (900 pulses at each frequency) or 100 Hz (four 1 sec trains delivered 20 sec apart). Following repetitive stimulation, fEPSP slopes were monitored for 1 h. Signals were amplified (Axoclamp 700B, Molecular Devices, recorded digitally (Digidata 1440A) and analyzed offline using Clampfit 10.

### Whole-cell patch clamp recordings from hippocampal slices

Visual patch recordings from CA1 pyramidal neurons were performed using the whole-cell configuration with holding potential at -60 mV. Patch pipettes, pulled from borosilicate glass (4-6 MΩ), were filled with an internal solution containing (in mM): Cs-gluconate 132.5, CsCl 17.5, HEPES 10, EGTA 0.2, Mg-ATP 2 and GTP 0.3 (pH 7.25, 290 mOsm). Synaptic responses were evoked with a concentric bipolar tungsten electrode located about 50 μm from the cell body layer in CA1. In some recordings, NMDAR-mediated EPSCs were pharmacology isolated by supplementing our aCSF with CNQX (10 μM). The AMPAR- and NMDAR-mediated EPSCs were measured at -70 mV and +40 mV, respectively. For NMDAR-EPSCs, the amplitude was measured 20 ms after the start of the stimulus artifact[21]. Signals were amplified using multiclamp 700B, sampled at 5 kHz, and analyzed with Clampfit 10 software (Molecular Devices).

### Western Blotting

For the basal protein expression and protein phosphorylation assays, hippocampal tissue from wild type and TRPM2^-/- ^mice (post-natal day 20-30) was used for Western blot. For experiments involving quinpirole treatment, hippocampal slices were incubated in ECS solution bubbled with 95%O2 and 5%CO2 at room temperature for at least 1 h, followed by exposing to quinpirole (10 μM) or vehicle for 30 min in aCSF. After 3 times wash with cold PBS, hippocampal tissue was homogenized in ice-cold RIPA buffer (50 mM Tris-HCl pH 7.4, 150 mM NaCl, 1 mM EDTA, 0.1% SDS, 0.5% Triton-X100, and 1% Sodium Deoxycholate) containing protease and phosphatase inhibitors. These samples were subjected to SDS-PAGE, transferred to a nitrocellulose membrane, which was then subjected to repeated stripping and successive probing with antibodies, as described. The signals were quantified using VersaDoc Imaging System (BioRad). The antibodies PSD95, pGSK3β^Ser9^, GSK3β, NR2A and NR2B were purchased from Cell Signaling Technology, and GluR1 and GluR2 from Santa Cruz Biotechnology, Inc.

### Statistics

Data are expressed as mean ± SEM. Statistical comparisons were made using either unpaired t-test or one-way ANOVA followed by Bonferroni *post-hoc *test, depending on the experimental protocol.

## Competing interests

The authors declare that they have no competing interests.

## Authors' contributions

YFX designed and performed extracellular field and whole-cell voltage-clamp recordings from hippocampal slices. JCB designed and performed whole-cell voltage-clamp recordings from cultured hippocampal neurons. GL designed and performed immunoblotting. YM generated TRPM2^-/- ^mice and contributed to the design of experiments. JFM, MT and MFJ conceptualized and supervised the project, contributed to the design of experiments and wrote the manuscript. All authors read and approved the final manuscript.

## Supplementary Material

Additional Files 1 **Short-term plasticity of excitatory synaptic transmission is unaltered in slices from TRPM2^-/-^**. Summary graph (a) and representative traces (b). Only a subset of interstimulus intervals are shown from a series recordings from WT (*n *= 11) and TRPM2^-/- ^(*n *= 11) slices. The time course for recovery from paired-pulse facilitation was identical in slices derived from WT and TRPM2^-/- ^mice. Click here for file
