# Supplementary figures and images for: Dependence of NMDA/GSK-3β Mediated Metaplasticity on TRPM2 Channels at Hippocampal CA3-CA1 Synapses
Source: Mol Brain. 2011 Dec 21;4:44. doi: 10.1186/1756-6606-4-44 (PMC3298512; doi:10.1186/1756-6606-4-44)

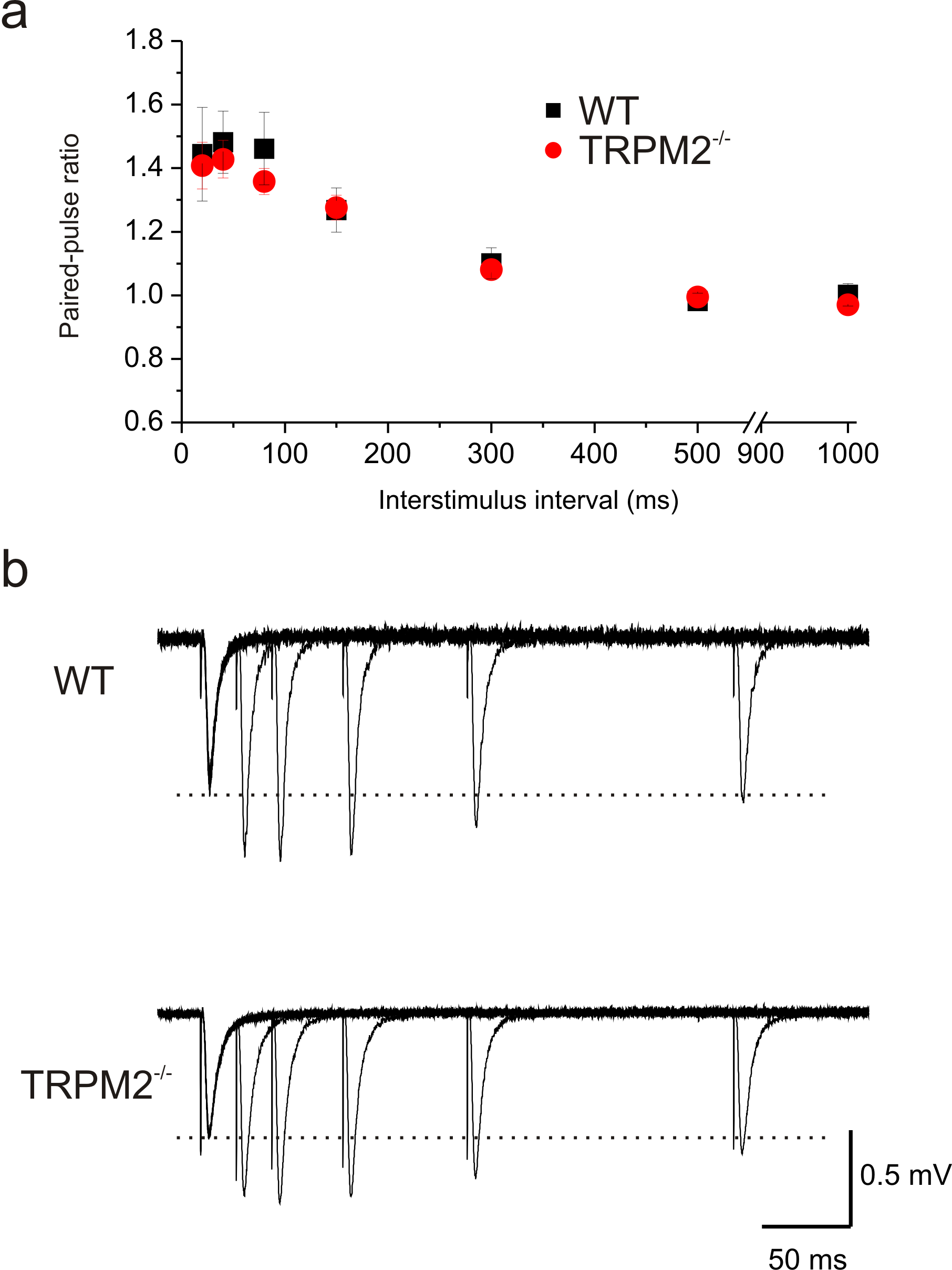

Supplement: Additional Files 1 — Short-term plasticity of excitatory synaptic transmission is unaltered in slices from TRPM2-/-. Summary graph (a) and representative traces (b). Only a subset of interstimulus intervals are shown from a series recordings from WT (n = 11) and TRPM2-/- (n = 11) slices. The time course for recovery from paired-pulse facilitation was identical in slices derived from WT and TRPM2-/- mice. [file 1756-6606-4-44-S1.TIFF]
